# Assessment of Scoring Balloons in STEMI Patients Treated With DCB‐Only Angioplasty: A Single Center Study

**DOI:** 10.1002/hsr2.70839

**Published:** 2025-05-21

**Authors:** Ioannis Merinopoulos, Natasha Corballis, Tharusha Gunawardena, U. Bhalraam, Rajkumar Natarajan, Johannes Reinhold, Upul Wickramarachchi, Clint Maart, Chris Sawh, Sreekumar Sulfi, Tim Gilbert, Trevor Wistow, Alisdair Ryding, Vassilios S. Vassiliou, Simon C. Eccleshall

**Affiliations:** ^1^ Department of Cardiology Norfolk and Norwich University Hospital Norwich Norfolk UK; ^2^ Norwich Medical School University of East Anglia Norwich UK

**Keywords:** DCB‐only angioplasty, scoring balloon, STEMI

## Abstract

**Background and Aims:**

A randomized trial has previously demonstrated that neointimal modification with a scoring balloon improves the anti‐restenotic effect of drug‐coated balloon (DCB) in patients with drug‐eluting stent restenosis. There are very limited data about the safety and efficacy of using scoring balloons as part of lesion preparation in patients with STEMI, especially in patients with de novo disease treated with DCB‐only angioplasty.

**Methods:**

We undertook an analysis of the SPARTAN Norwich Registry to address this question. We compared the composite endpoint of cardiovascular mortality or unplanned target lesion revascularization in the DCB‐only cohort stratified based on the use or not of scoring balloon as part of the lesion preparation. Furthermore, we undertook a propensity score‐matched analysis of the DCB‐only cohort.

**Results:**

A total of 452 consecutive patients were treated with DCB‐only angioplasty and scoring balloon was used in 121 patients as part of the lesion preparation. Scoring balloon was not a significant predictor of the composite endpoint even after propensity score‐matched analysis. Chronic obstructive pulmonary disease was the only significant predictor of the composite endpoint after propensity score‐matched analysis.

**Conclusion:**

This is the first study demonstrating the safety and efficacy of scoring balloon as part of lesion preparation in patients with STEMI due to de novo disease treated with DCB‐only angioplasty.

**Trial Registration:**

https://clinicaltrials.gov/ct2/show/NCT04482972; Unique identifier: NCT04482972.

- **What is known?**
  ∘ Scoring balloon improves efficacy of drug‐coated balloons when treating drug‐eluting‐stent in‐stent restenosis.
- **What is new?**
  ∘ Scoring balloon is safe and efficacious in patients with STEMI due to de novo disease being treated with drug‐coated balloon.
- **What is the clinical implication?**
  ∘ Scoring balloon may be used as part of lesion preparation in patients with STEMI due to de novo disease being treated with drug‐coated balloon.

## 1 Introduction

There are very limited data about the use of scoring balloon in patients with ST elevation myocardial infarction (STEMI), especially in patients with de novo disease treated with drug‐coated balloon (DCB)‐only angioplasty. The ISAR‐DESIRE 4 trial has previously demonstrated that neointimal modification with scoring balloon improves the anti‐restenotic efficacy of DCB in patients presenting with drug‐eluting stent (DES) in‐stent restenosis [1]. The more recent SCRAP study, which exclusively used a scoring balloon for lesion preparation, demonstrated the safety and efficacy of a stent‐minimization strategy in an all‐comer population with de novo disease [2]. However, only 11% of the DCB‐only group in the SCRAP study had presented with STEMI making it difficult to draw definitive conclusions about patients with STEMI (2).

Our group recently demonstrated the safety and efficacy of DCB‐only angioplasty in STEMI due to de novo disease, compared with 2nd generation DES (3). The aim of this analysis was to assess the safety and efficacy of scoring balloon use in DCB in the SPARTAN Norwich Registry [3], as part of lesion preparation, in patients with STEMI due to de novo disease treated with DCB‐only angioplasty.

## 2 Methods

The assessment of scoring balloons in STEMI treated with DCB‐only angioplasty was an investigator‐initiated single‐center, specific analysis of the SPARTAN Norwich STEMI Registry. The STEMI analysis has already been published, and the detailed methodology of our previous study is available [4]. In brief, it included patients with STEMI due to de novo disease treated either with DCB‐only angioplasty or 2nd generation DES. Patients with cardiac arrest, intubation, or cardiogenic shock were excluded. For the current subgroup analysis, the database was interrogated to identify patients where scoring balloon was used as part of lesion preparation. The number of pre‐dilatation balloons as well as the order (1st, 2nd, 3rd balloon) of scoring balloon use was identified from the database. Patient outcomes including mortality, cardiovascular mortality, acute coronary syndrome, stroke, major bleeding, and unplanned target lesion revascularization (TLR) were already available from the previous study. For the current analysis, the primary endpoint was the composite of cardiovascular mortality or unplanned TLR. This is a device‐orientated endpoint aiming to provide an assessment of the safety and efficacy of use of scoring balloon in patients with STEMI due to de novo disease treated with DCB‐only angioplasty [5].

An independent data scientist undertook the statistical analysis using R version 4.3.2 (R Language and Environment for Statistical Computing) on RStudio version 2023.12.0 (Posit Software, PBC). Nominal/Ordinal variables were reported as count (percentage). Normality of distribution of continuous variables was assessed by the Kolmogorov–Smirnov test. Normally distributed variables were presented as mean (standard deviation) whilst non‐normally distributed variables were presented as median (interquartile range). Depending on the type of variable, Pearson's Chi‐squared test, Wilcoxon's rank sum test, and Fisher's exact test were used to determine differences between groups. We established a significance threshold of 0.05.

In the event where significant differences were observed between those who had scoring balloon used as part of lesion preparation and those without, the research team decided to adopt propensity score matching to account for these differences. The “matchit” package [6] for R was used to undertake propensity score matching. Univariate Cox Regression analysis and table comparisons were used to determine the variables to match age, hypertension, peripheral vascular disease, cerebrovascular events, myocardial infarctions, heart failure, atrial fibrillation, family history of coronary artery disease, chronic obstructive pulmonary disease, diabetes mellitus, estimated glomerular filtration rate, treated vessel, bifurcation disease, frailty score, heavy calcification, and acuity score. The “optimal” 1:1 matching method without replacement was specified for this process.

Cumulative hazard plots were generated for the primary end point. For each plot, the displayed *p* value shown is generated by the log‐rank test. Mixed effects Cox regression multivariate models were used to generate the multivariate analysis. This model was chosen as it allows to correct for the biasness that typically accumulate with matching. Schoenfeld residuals were calculated for each regression variable to verify that the assumptions of the Cox model are upheld (Central Illustration Illustration 1, 1).

**Central Illustration 1:**
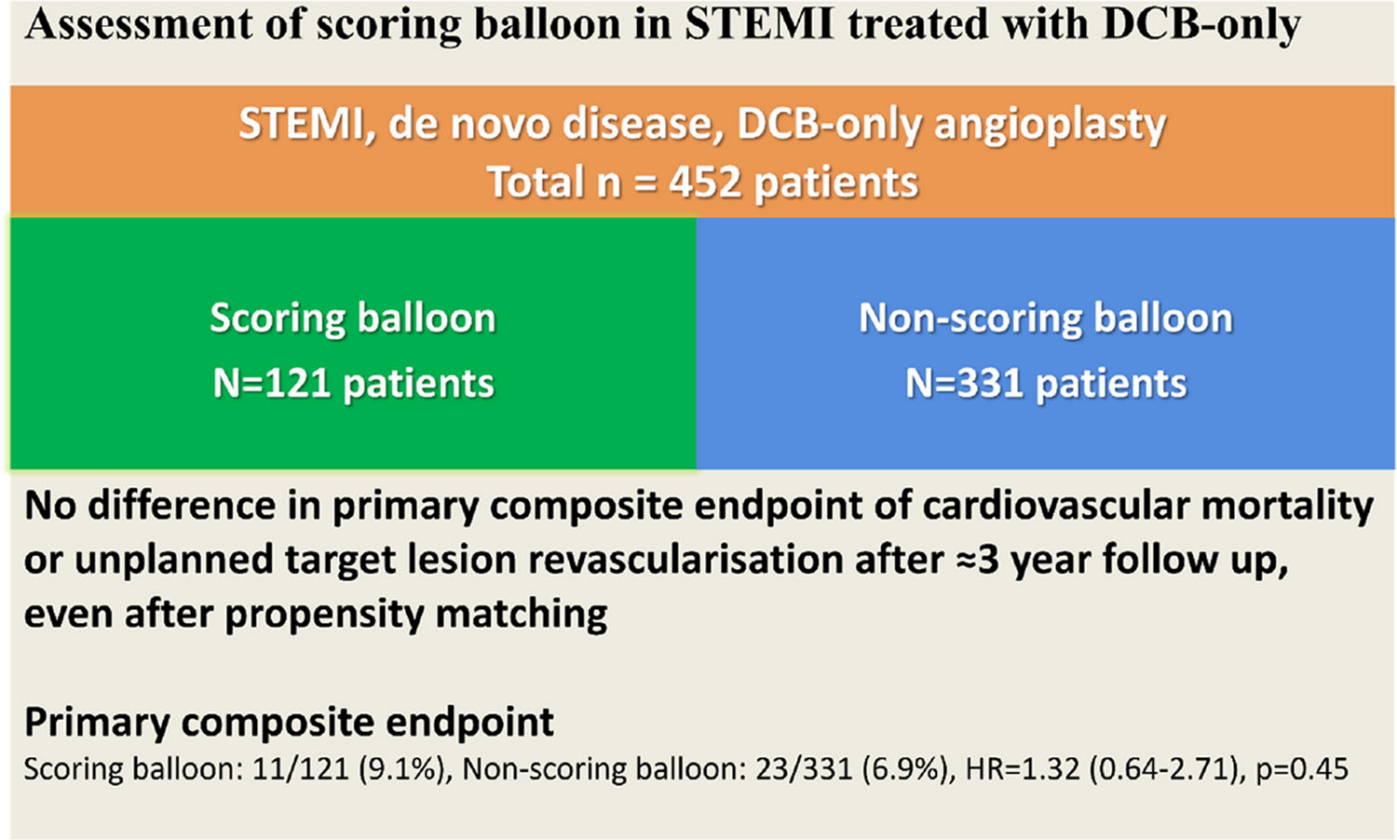
Central illustration demonstrating the main findings of our study.

## 3 Results

As shown in Figure Illustration 1, 1, 452 consecutive patients were treated with DCB‐only angioplasty and 687 consecutive patients with 2nd generation DES. In the DCB‐only cohort, 121 patients (27%) were treated with a scoring balloon as part of their lesion preparation, while 331 patients were not. All DCBs used were SeQuent Please NEO (B. Braun). Out of the 121 patients, 68 patients (56.2%) had noncompliant Scoreflex balloon (OrbusNeich) used, while 53 (43.8%) had an NSE Alpha balloon (Nipro) used. Only 35 patients (5%) in the DES‐only cohort were treated with a scoring balloon. There were significant differences within the DCB‐only cohort between the scoring and no‐scoring groups. The scoring balloon group had significantly more patients with left main stem (LMS) or left anterior descending (LAD) disease, heavily calcified vessels and larger mean vessel diameter. In the scoring‐balloon group, 2.35 ± 0.7 pre‐dilatation balloons were used vs. 1.79 ± 0.66 in the no‐scoring balloon group (*p* < 0.001). Furthermore, the fluoroscopy time was significantly longer in the scoring balloon group [10.4 (7.1–14.1) vs. 8.6 (6.4–11.4); *p* = 0.01] (Table 1).

**Figure 1:**
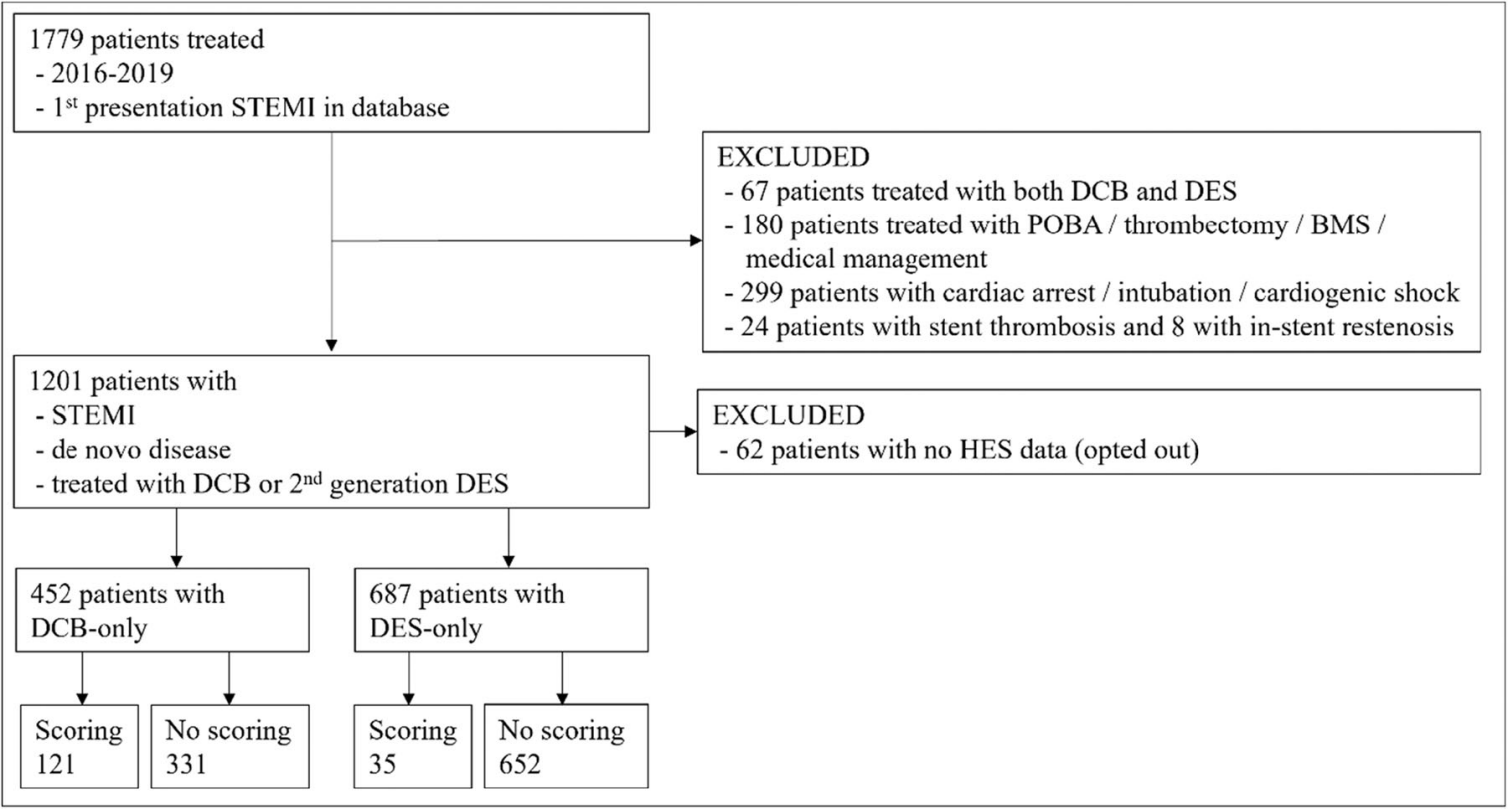
Flow diagram of patients in the study.

**Table 1:** Baseline clinical and angiographic characteristics.

	DCB group		
	Scoring balloon	No‐scoring balloon	*p* value
Patients	121	331	
Age	64.7 (± 13.4)	66.1 (± 12.2)	0.15
Male	89 (73.6)	241 (72.8)	0.87
DCB	121 (100)	331 (100)	n/a
Hypercholesterolemia	20 (16.5)	59 (17.8)	0.75
Hypertension	58 (47.9)	125 (37.8)	0.051
Peripheral vascular disease	2 (1.7)	5 (1.5)	0.91
Stroke	5 (4.1)	13 (3.9)	0.92
Myocardial infarction	8 (6.6)	22 (6.6)	0.98
PCI	5 (4.1)	20 (6)	0.43
CABG	1 (0.8)	4 (1.2)	0.73
Atrial fibrillation	13 (10.7)	24 (7.3)	0.23
Family history of IHD	9 (7.4)	35 (10.6)	0.32
COPD	5 (4.1)	22 (6.6)	0.32
Diabetes	15 (12.4)	48 (14.5)	0.57
Smoking	70 (57.9)	184 (55.6)	0.78
eGFR	92.7 ± 25.4	91.3 ± 27.6	0.31
Frailty	4 (3.3)	12 (3.6)	0.87
LMS/LAD	63 (52.1)	135 (40.8)	**0.03**
LMS	2 (1.7)	0	**0.02**
Multivessel PCI	5 (4.1)	12 (3.6)	0.78
Mean vessel diameter	3.41 ± 0.47	3.24 ± 0.56	**0.001**
Lesion length	30.5 ± 13.9	28.7 ± 12.5	0.09
Vessel diameter > 3 mm	110 (90.9)	253 (76.4)	**< 0.001**
Bifurcation	51 (42.1)	137 (41.4)	0.88
True bifurcation	12 (9.9)	38 (11.5)	0.64
Severe calcification	27 (22.3)	40 (12.1)	**0.007**
Diffuse disease	26 (21.5)	95 (28.7)	0.12
Tortuosity	21 (17.4)	41 (12.4)	0.18
Fluoroscopy time (min)	10.4 (7.1–14.1)	8.6 (6.4–11.4)	**0.013**
Contrast volume (mL)	120 (100–150)	110 (100–140)	0.11
PRE‐PCI TIMI flow			
TIMI 0–1	92 (76)	239 (72)	0.44
TIMI 2–3	29 (24)	92 (28)	
POST‐PCI TIMI flow			
TIMI 0–1	1 (0.8)	3 (0.9)	> 0.99
TIMI 2–3	120 (99)	328 (99)	
Coronary dissections at end of PCI			0.34
No angiographic evidence	81 (66.9)	237 (71.6)	
Type A	18 (14.9)	54 (16.3)	
Type B	22 (18.2)	40 (12.1)	
Acuity score	16 (12–23)	18 (14–23)	0.21

*Note:* Bold values indicate statistically significant *p* < 0.05.

Abbreviations: CABG, coronary artery bypass grafting; DCB, drug‐coated balloon; DES, drug‐eluting stent; eGFR, estimated glomerular filtration rate; IHD, ischemic heart disease; LAD, left anterior descending; LMS, left main stem; PCI, percutaneous coronary intervention.

Figure S1 demonstrates the distribution of number of pre‐dilatation balloons in the scoring and no‐scoring groups. One balloon was used in 9.9% of scoring group vs. 33.8% of no‐scoring group; two balloons were used in 61.2% of scoring group vs. 51.7% of no‐scoring group; and three balloons were used in 33.1% of scoring group vs. 12.4% of no‐scoring group.

Figure S2 demonstrates the additional balloons used after scoring balloon. It shows that in all but one case, the scoring balloon was either the last or second to last balloon used.

**Figure 2:**
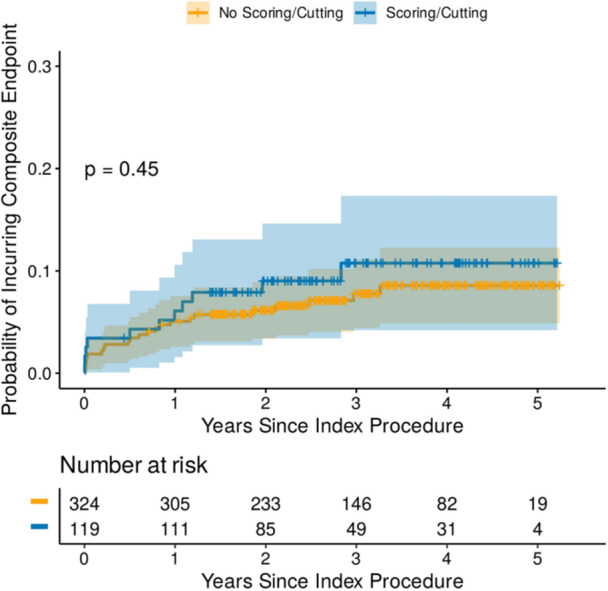
Cumulative hazard estimator plot for composite endpoint in DCB cohort.

After a mean follow‐up of 1063 ± 478 days, 11 patients (9.1%) suffered a cardiovascular death or had an unplanned TLR in the scoring balloon group vs. 23 patients (6.9%) in the no‐scoring balloon group (*p* = 0.45). Univariable Cox regression analysis demonstrated that use of scoring balloon was not a significant predictor of the primary outcome (HR = 1.32, CI = 0.64–2.71, *p* = 0.45). Table S1 indicates the univariable Cox regression analysis in the DCB cohort. Cumulative hazard estimator plot demonstrated no difference in terms of the composite endpoint with use of scoring balloon in the DCB cohort (Figure 2). The rate of the components of the primary endpoint for scoring balloon vs. no‐scoring balloon group were as follows: CV mortality (8.3% vs. 4.2%) and TLR (0.8% vs. 2.8%) (Figures S3 and S4). There was also no difference in terms of the composite endpoint of cardiovascular death, acute coronary syndrome, or TLR (Figure S5)

**Figure 3:**
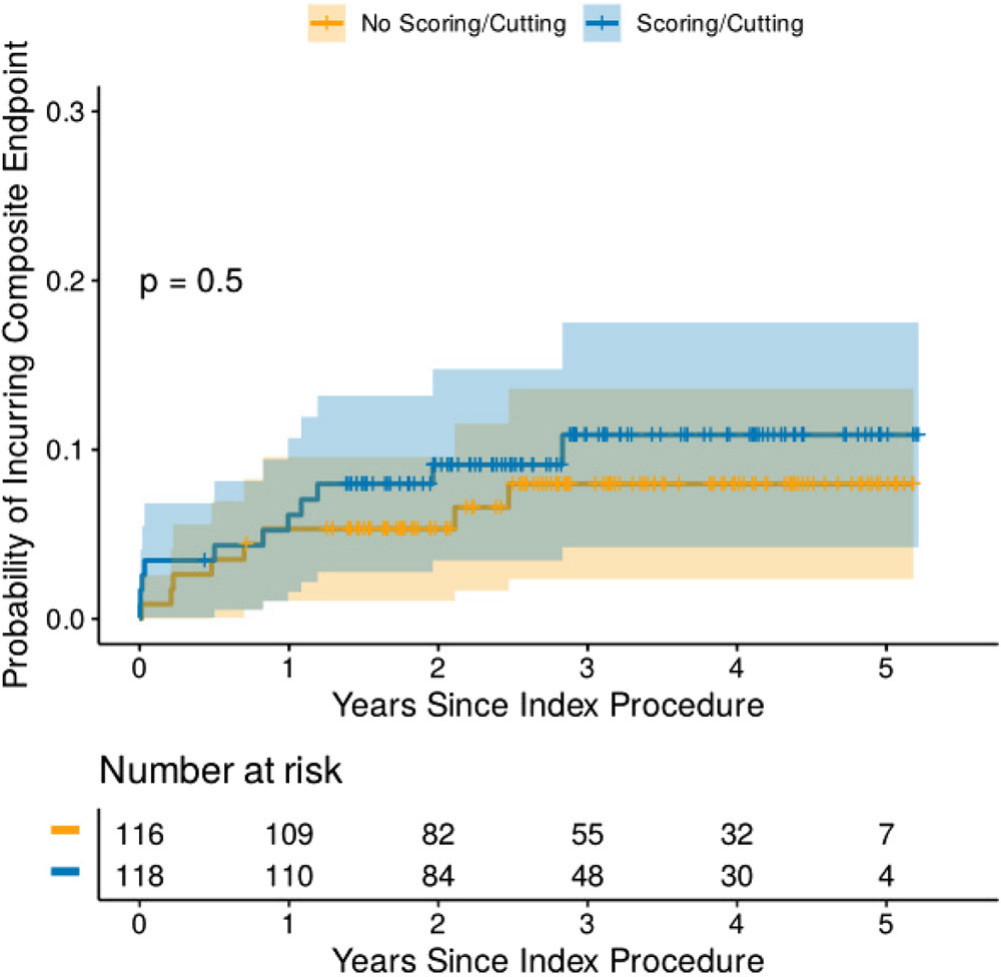
Cumulative hazard estimator plot for composite endpoint in DCB cohort following propensity score matching.

As there were significant differences between scoring and no‐scoring groups in the DCB‐only cohort, we performed a propensity score‐matched analysis matching 120 patients treated with DCB and a scoring balloon to 120 patients treated with DCB but without a scoring balloon. The baseline patient and angiographic characteristics of the propensity‐matched DCB cohort are demonstrated in Table S2. Multivariable Cox regression analysis of the propensity‐matched DCB cohort indicated that COPD was the only independent predictor of the composite endpoint (Table 2). The cumulative hazard estimator plot demonstrated no significant difference in terms of the composite endpoint, between scoring and no‐scoring balloon following propensity score matching as well (Figure 3). The rate of the components of the primary endpoint for scoring vs. no‐scoring balloon groups was as follows: CV mortality (8.3% vs. 1.7%) and TLR (0.8% vs. 4.2%) (Figures S6 and S7). Further analysis demonstrated that there was no difference in terms of the composite endpoint of cardiovascular death, acute coronary syndrome, or TLR (Figure S8).

**Table 2:** Multivariable Cox regression analysis.

Variable	HR (95% CI)	*p* value
Scoring balloon	0.71 (0.33–1.52)	0.37
Age	1.03 (1.00–1.07)	0.10
Peripheral vascular disease	4.07 (0.41–40.1)	0.23
Stroke	1.66 (0.26–10.5)	0.59
Myocardial infarction	2.43 (0.81–7.34)	0.11
COPD	4.33 (1.24–15.1)	**0.02**
eGFR	1.00 (0.98–1.02)	0.98
True bifurcation	0.65 (0.17–2.54)	0.54
Frailty score	1.05 (0.78–1.40)	0.76
Heavy calcification	1.48 (0.63–3.49)	0.37

*Note:* Bold values indicate statistically significant *p* < 0.05.

Abbreciations: CI, confidence interval; COPD, chronic obstructive pulmonary disease; eGFR, estimated glomerular filtration rate.

## 4 Discussion

This is the largest cohort analysis assessing the safety of scoring balloon in patients with STEMI due to de novo disease treated with DCB‐only angioplasty. It showed no difference between scoring and no‐scoring balloon as part of lesion preparation, in terms of the composite endpoint of cardiovascular mortality or unplanned TLR even after propensity score‐matched analysis (visual overview).

Our group has previously demonstrated the safety and efficacy of DCB‐only angioplasty compared to 2nd generation DES in patients with STEMI due to de novo disease [4, 7]. Patients with STEMI present particular challenges such as vasoconstriction and high clot burden which make lesion preparation more demanding. Use of scoring balloons might be necessary as part of lesion preparation to achieve optimal angioplasty results, especially if the treatment strategy is DCB‐only angioplasty. Achievement of adequate luminal gain with < 30% recoil and no more than type B dissections are the recommended pre‐requisites before DCB angioplasty [8, 9, 10]. Use of these specialized balloons can be a matter of concern amongst interventional cardiologists especially if they are used in high‐risk patients, such as patients with STEMI, and if the treatment strategy is DCB‐only angioplasty without stent to cover potential coronary dissections [11, 12].

Our study has demonstrated that use of scoring balloon is safe in a large cohort of consecutive patients with STEMI due to de novo disease being treated with DCB‐only angioplasty. Even though there was angiographic evidence of coronary dissections in 30% of patients, there was no significant difference between scoring and no‐scoring balloon groups in terms of dissections. As expected, the scoring balloon group had significantly more patients with heavily calcified vessels and necessitated significantly more pre‐dilatation balloons, indicating more complicated lesions where further plaque and calcium modification was necessary to achieve optimal lesion preparation. Our results are consistent and complimentary with the recent SCRAP study, which used exclusively scoring balloon for lesion preparation and demonstrated the safety of a stent‐minimization strategy in an all‐comer population. However, the SCRAP study included only 11% of patients with STEMI making it difficult to draw definitive conclusions from this study alone [2].

The ISAR‐DESIRE4 is the only randomized trial that has assessed the effect of scoring balloon before DCB treatment. It demonstrated that in the context of DES‐ISR, scoring balloon as assessed by quantitative coronary angiography, provides superior neointimal modification compared to standard balloon dilatation and improves the anti‐restenotic effect of DCB (1). There are only limited data about the use of scoring balloon before DCB‐only angioplasty for de novo disease, a setting which requires adequate lesion expansion without flow‐limiting dissections [13, 14]. A recent retrospective study demonstrated that use of scoring balloon was an independent predictor of optimal angiographic result, and it was associated with decreased risk of severe dissection compared to a nonscoring balloon [14]. The results of our study showed no significant difference in terms of coronary dissections between the scoring and no‐scoring groups. The most likely reason that our study did not demonstrate decreased risk of dissections in the scoring balloon group, is the liberal use of other pre‐dilatation balloons in both groups. Our wider experience suggests that scoring balloon increases the probability of achieving optimal lesion preparation without increasing the risk of high‐grade dissection therefore allows more use of DCB as a treatment strategy.

Previous data have demonstrated that scoring/cutting balloons limit the degree of vascular injury and elicited inflammation by directing the force to the scoring/cutting elements as compared to the blunt trauma of simple semi‐ or noncompliant balloons [15]. Funatsu et al. had demonstrated previously that type B dissections are associated with a larger net gain when compared to type A or no dissections, while type D dissections were significantly associated with restenosis [16]. Consistent with these results, a recent prospective study, that used exclusively scoring/cutting balloons for lesion preparation under intravascular guidance, demonstrated that the dissection index was significantly associated with lumen and vessel enlargement, and it was also the strongest predictor of future late lumen enlargement [17].

In summary, the limited available data support the concept that scoring balloons can facilitate optimal lesion preparation via means of controlled dissections and limited vascular injury. Our study has demonstrated that their use is safe even in patients with STEMI due to de novo disease treated with DCB‐only angioplasty.

### 4.1 Limitations

Our study has some limitations, as it is a subgroup analysis of a retrospective non‐randomized study from a single center. However, we have included all consecutive patients who met the inclusion criteria minimizing selection bias. The decision to use a scoring balloon or not was left up to the discretion of the operator who used what they believed would provide the best results. Therefore, as this decision was not randomized it is a study limitation. We have tried to ameliorate that by performing a propensity score‐matched analysis of the DCB‐only cohort. In addition, the fact that the scoring balloon was either the last or second to last balloon used, indicates that they were used for undilatable or difficult to treat lesions with excessive recoil. Therefore, this represents a bias of our study. Furthermore, we do not have data on patients who required bailout stenting due to high‐grade dissection during the lesion preparation, detailed procedural data such as quantititative coronary angiography or data on left ventricular ejection fraction. Intravascular imaging was used only in a minority of cases, and there are no available data on peak troponin or creatinine kinase. Lastly, as it is a retrospective analysis, we have not performed a formal power calculation, but we have included all patients meeting our inclusion criteria.

## 5 Conclusion

This is the largest propensity score‐matched study demonstrating the safety of scoring balloon as part of lesion preparation before DCB‐only angioplasty for de novo disease in patients with STEMI.

## Author Contributions

**Ioannis Merinopoulos:** conceptualization, data curation, formal analysis, methodology, writing – original draft. **Natasha Corballis:** conceptualization, writing – review and editing. **Tharusha Gunawardena:** writing – review and editing. **U. Bhalraam:** formal analysis, writing – review and editing. **Rajkumar Natarajan:** writing – review and editing. **Johannes Reinhold:** writing – review and editing. **Upul Wickramarachchi:** writing – review and editing. **Clint Maart:** writing – review and editing. **Chris Sawh:** writing – review and editing. **Sreekumar Sulfi:** writing – review and editing. **Tim Gilbert:** writing – review and editing. **Trevor Wistow:** writing – review and editing. **Alisdair Ryding:** writing – review and editing. **Vassilios S. Vassiliou:** methodology, writing – review and editing. **Simon C. Eccleshall:** conceptualization, methodology, writing – review and editing.

## Ethics Statement

The study was approved by the institutional review boards and the Norfolk & Norwich University Hospital and Northwest Haydock research ethics committee.

## Consent

Not deemed necessary according to Confidentiality Advisory Group (17/CAG/0145).

## Conflicts of Interest

Ioannis Merinopoulos has received research grant from Cordis. Vassilios S. Vassiliou has received research grant from B Braun. Simon C. Eccleshall has received research grant and honoraria from B Braun and honoraria from Cordis/Medalliance. The other authors have nothing relevant to declare. The supporting financial sources had no involvement in study design, collection, analysis, interpretation of data, writing the report or decision to submit for publication.

## Transparency Statement

The lead author Ioannis Merinopoulos affirms that this manuscript is an honest, accurate, and transparent account of the study being reported; that no important aspects of the study have been omitted; and that any discrepancies from the study as planned (and, if relevant, registered) have been explained.

## Supporting information

Supporting figure 1.

Supporting figure 2.

Supporting figure 3.

Supporting figure 4.

Supporting figure 5.

Supporting figure 6.

Supporting figure 7.

Supporting figure 8.

Supporting table 1.

Supporting table 2.

## Data Availability

Data can be made available following an appropriate request to the authors.

## References

[1] S. Kufner , M. Joner , S. Schneider , et al., “Neointimal Modification With Scoring Balloon and Efficacy of Drug‐Coated Balloon Therapy in Patients With Restenosis in Drug‐Eluting Coronary Stents,” JACC: Cardiovascular Interventions 10 (2017): 1332–1340. 28683939 10.1016/j.jcin.2017.04.024

[2] L. Meunier , M. Godin , G. Souteyrand , et al., “Prospective, Single‐Centre Evaluation of the Safety and Efficacy of Percutaneous Coronary Interventions Following a Decision Tree Proposing a No‐Stent Strategy in Stable Patients With Coronary Artery Disease (SCRAP Study),” Clinical Research in Cardiology 112 (2023): 1164–1174. 35776144 10.1007/s00392-022-02054-7 PMC10449686

[3] DCBNORWICH, *Health Research Authority*, n.d., accessed 18 June 2024, https://www.hra.nhs.uk/planning-and-improving-research/application-summaries/research-summaries/dcbnorwich/.

[4] I. Merinopoulos , T. Gunawardena , N. Corballis , et al., “Assessment of Paclitaxel Drug Coated Balloon Only Angioplasty in STEMI,” JACC: Cardiovascular Interventions 16, no. 7 (2023): 771–779. 37045498 10.1016/j.jcin.2023.01.380

[5] H. M. Garcia‐Garcia , E. P. McFadden , A. Farb , et al., “Standardized End Point Definitions for Coronary Intervention Trials: The Academic Research Consortium‐2 Consensus Document,” Circulation 137 (2018): 2635–2650. 29891620 10.1161/CIRCULATIONAHA.117.029289

[6] D. E. Ho , K. Imai , G. King , and E. A. Stuart , MatchIt: Nonparametric Preprocessing for Parametric Causal Inference, Journal of Statistical Software, n.d.

[7] I. Merinopoulos , T. Gunawardena , U. Wickramarachchi , et al., “Long‐Term Safety of Paclitaxel Drug‐Coated Balloon‐Only Angioplasty for De Novo Coronary Artery Disease: The SPARTAN DCB Study,” Clinical Research in Cardiology 110 (2021): 220–227. 32876814 10.1007/s00392-020-01734-6 PMC7862512

[8] R. V. Jeger , S. Eccleshall , W. A. Wan Ahmad , et al., “Drug‐Coated Balloons for Coronary Artery Disease,” JACC: Cardiovascular Interventions 13 (2020): 1391–1402. 32473887 10.1016/j.jcin.2020.02.043

[9] C. Yerasi , B. C. Case , B. J. Forrestal , et al., “Drug‐Coated Balloon for De Novo Coronary Artery Disease,” Journal of the American College of Cardiology 75 (2020): 1061–1073. 32138967 10.1016/j.jacc.2019.12.046

[10] D. Giacoppo , J. Saucedo , and B. Scheller , “Coronary Drug‐Coated Balloons for De Novo and In‐Stent Restenosis Indications,” Journal of the Society for Cardiovascular Angiography & Interventions 2 (2023): 100625. 39130710 10.1016/j.jscai.2023.100625 PMC11308150

[11] A. Colombo and P. P. Leone , “Redefining the Way to Perform Percutaneous Coronary Intervention: A View in Search of Evidence,” European Heart Journal 44 (2023): 4321–4323. 37038750 10.1093/eurheartj/ehad215

[12] Z. Rahman , M. Ullah , and A. Choudhury , “Coronary Artery Dissection and Perforation Complicating Percutaneous Coronary Intervention: A Review,” Cardiovascular Journal 3 (2011): 239–247.

[13] S. Basavarajaiah , V. Sharma , D. Agip‐Fustamante , et al., “Which Predilatation Balloons Provide the Best Lesion Preparation Prior to Use of Drug Coated Balloons in De‐Novo Lesions? Results From the PREPARE Study,” Minerva Cardiology and Angiology 71, no. 2 (2023): 182–188, 10.23736/S2724-5683.22.05989-0. 35420280

[14] E.‐S. Shin , S. H. Ann , M. H. Jang , et al., “Impact of Scoring Balloon Angioplasty on Lesion Preparation for DCB Treatment of Coronary Lesions,” Journal of Clinical Medicine 12 (2023): 6254. 37834898 10.3390/jcm12196254 PMC10573989

[15] T. Inoue , Y. Sakai , K. Hoshi , I. Yaguchi , T. Fujito , and S. Morooka , “Lower Expression of Neutrophil Adhesion Molecule Indicates Less Vessel Wall Injury and Might Explain Lower Restenosis Rate After Cutting Balloon Angioplasty,” Circulation 97 (1998): 2511–2518. 9657471 10.1161/01.cir.97.25.2511

[16] A. Funatsu , T. Kobayashi , M. Mizobuchi , and S. Nakamura , “Clinical and Angiographic Outcomes of Coronary Dissection After Paclitaxel‐Coated Balloon Angioplasty for Small Vessel Coronary Artery Disease,” Cardiovascular Intervention and Therapeutics 34 (2019): 317–324. 30652250 10.1007/s12928-019-00571-3

[17] T. Yamamoto , T. Sawada , K. Uzu , T. Takaya , H. Kawai , and Y. Yasaka , “Possible Mechanism of Late Lumen Enlargement After Treatment for De Novo Coronary Lesions With Drug‐Coated Balloon,” International Journal of Cardiology 321 (2020): 30–37. 32710988 10.1016/j.ijcard.2020.07.028

