# Supplementary material for: Assessment of Scoring Balloons in STEMI Patients Treated With DCB‐Only Angioplasty: A Single Center Study
Source: Health Sci Rep. 2025 May 21;8(5):e70839. doi: 10.1002/hsr2.70839 (PMC12095844; doi:10.1002/hsr2.70839)
Supplement: Supplementary file 2 — Supporting figure 2. [file HSR2-8-e70839-s006.docx]

Supplementary figure 2: Additional balloons used after scoring balloons
