# Supplementary material for: Assessment of Scoring Balloons in STEMI Patients Treated With DCB‐Only Angioplasty: A Single Center Study
Source: Health Sci Rep. 2025 May 21;8(5):e70839. doi: 10.1002/hsr2.70839 (PMC12095844; doi:10.1002/hsr2.70839)

Supplementary figure 6: cumulative hazard estimator plot for cardiovascular mortality in propensity score matched cohort


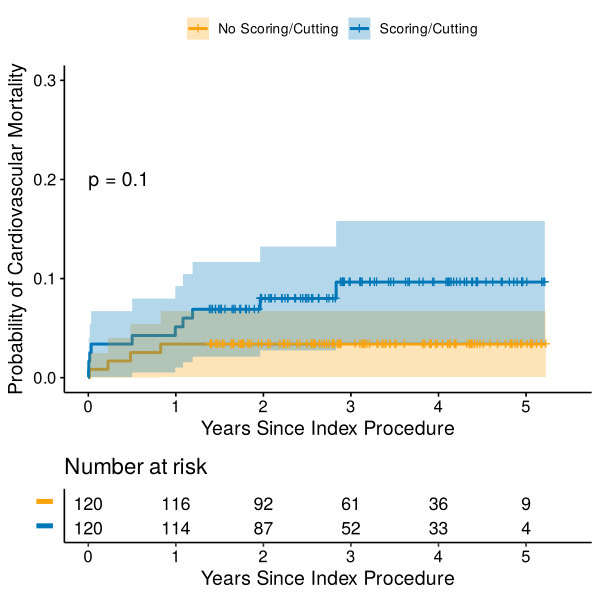

Supplement: Supplementary file 6 — Supporting figure 6. [file HSR2-8-e70839-s002.docx]
