# Supplementary material for: Assessment of Scoring Balloons in STEMI Patients Treated With DCB‐Only Angioplasty: A Single Center Study
Source: Health Sci Rep. 2025 May 21;8(5):e70839. doi: 10.1002/hsr2.70839 (PMC12095844; doi:10.1002/hsr2.70839)

Supplementary figure 8: cumulative hazard estimator plot for composite endpoint of cardiovascular death, acute coronary syndrome or target lesion revascularisation in propensity score matched cohort


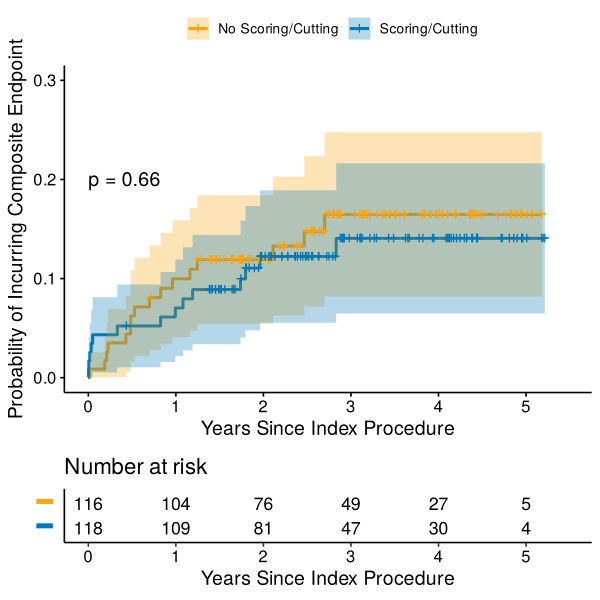

Supplement: Supplementary file 8 — Supporting figure 8. [file HSR2-8-e70839-s001.docx]
