# Supplementary material for: Assessment of Scoring Balloons in STEMI Patients Treated With DCB‐Only Angioplasty: A Single Center Study
Source: Health Sci Rep. 2025 May 21;8(5):e70839. doi: 10.1002/hsr2.70839 (PMC12095844; doi:10.1002/hsr2.70839)
Supplement: Supplementary file 9 — Supporting table 1. [file HSR2-8-e70839-s004.docx]

Supplementary table 1: Univariable Cox regression analysis for the composite endpoint in the DCB cohort

| Variable | HR (95%CI) | P value |
| --- | --- | --- |
| Scoring balloon | 1.32 (0.64 – 2.71) | 0.45 |
| Age | 1.03 (1.01 – 1.06) | **0.017** |
| Female sex | 0.96 (0.45 – 2.05) | 0.91 |
| Hypercholesterolaemia | 0.61 (0.22 – 1.74) | 0.36 |
| Hypertension | 1.04 (0.52 – 2.05) | 0.92 |
| Peripheral vascular disease | 4.31 (1.03 – 18) | **0.045** |
| Stroke | 3.45 (1.21 – 9.8) | **0.02** |
| Myocardial infarction | 2.70 (1.04 – 6.98) | **0.041** |
| PCI | 2.53 (0.89 – 7.20) | 0.081 |
| CABG | 0 (0 – inf) | 0.99 |
| Atrial fibrillation | 1.53 (0.54 – 4.34) | 0.42 |
| Family history of ischaemic heart disease | 0.85 (0.26 – 2.78) | 0.79 |
| Chronic obstructive pulmonary disease | 3.25 (1.26 – 8.39) | **0.015** |
| Diabetes | 0.83 (0.29 – 2.35) | 0.72 |
| Smoking history | 0.95 (0.48 – 1.90) | 0.89 |
| Estimated glomerular filtration rate | 0.99 (0.97 – 1.00) | **0.045** |
| Frailty | 1.18 (1.05 – 1.33) | **0.005** |
| Left main stem treated | 15.6 (3.72 – 65.3) | **<0.001** |
| Left main stem/Left anterior descending artery treated | 1.80 (0.91 – 3.56) | 0.092 |
| Multivessel percutaneous coronary intervention | 1.72 (0.41 – 7.17) | 0.46 |
| Vessel diameter | 1.33 (0.72 – 2.45) | 0.36 |
| Lesion length | 1.01 (0.98 – 1.05) | 0.39 |
| Vessel diameter >3mm | 2.50 (0.76 – 8.17) | 0.13 |
| Bifurcation disease | 1.12 (0.57 – 2.20) | 0.75 |
| True bifurcation | 3.54 (1.69 – 7.41) | **<0.001** |
| Heavy calcification | 3.30 (1.63 – 6.66) | **<0.001** |
| Acuity score | 1.06 (1.01 – 1.11) | **0.016** |
| Fluoroscopy time (min) | 1.00 (0.98 – 1.01) | 0.85 |
| Contrast volume (ml) | 1.00 (1.00 to 1.01) | 0.30 |

Supplementary table 1 demonstrates the univariable Cox regression analysis for the composite endpoint in the DCB cohort
