# Supplementary material for: Assessment of Scoring Balloons in STEMI Patients Treated With DCB‐Only Angioplasty: A Single Center Study
Source: Health Sci Rep. 2025 May 21;8(5):e70839. doi: 10.1002/hsr2.70839 (PMC12095844; doi:10.1002/hsr2.70839)
Supplement: Supplementary file 10 — Supporting table 2. [file HSR2-8-e70839-s009.docx]

Supplementary table 2: Baseline patient and angiographic characteristics of the propensity matched DCB cohort

| Characteristic | Overall (N=240) | No scoring balloon (N=120) | Scoring balloon (N=120) | P value |
| --- | --- | --- | --- | --- |
| Age, median (IQR) | 65 (55 – 74) | 64 (56 – 74) | 66 (54 – 74) | 0.94^1^ |
| Male | 176 (73%) | 88 (73%) | 88 (73%) | >0.99^3^ |
| Hypercholesterolaemia | 42 (18%) | 22 (18%) | 20 (17%) | 0.73^3^ |
| Hypertension | 114 (48%) | 56 (47%) | 58 (48%) | 0.80^3^ |
| PVD | 2 (0.8%) | 0 (0.0%) | 2 (1.7%) | >0.50^2^ |
| Stroke | 9 (3.8%) | 4 (3.3%) | 5 (4.2%) | >0.99^2^ |
| Myocardial infarction | 17 (7.1%) | 9 (7.5%) | 8 (6.7%) | 0.80^3^ |
| PCI | 11 (4.6%) | 6 (5.0%) | 5 (4.2%) | >0.76^3^ |
| CABG | 3 (1.3%) | 2 (1.7%) | 1 (0.8%) | >0.99^2^ |
| Atrial fibrillation | 19 (7.9%) | 6 (5.0%) | 13 (11%) | 0.09^3^ |
| Family history of IHD | 18 (7.5%) | 9 (7.5%) | 9 (7.5%) | >0.99^3^ |
| COPD | 8 (3.3%) | 3 (2.5%) | 5 (4.2%) | 0.72^2^ |
| Diabetes | 26 (11%) | 12 (10%) | 14 (12%) | 0.68^3^ |
| Smoking status | 133 (57%) | 63 (53%) | 70 (60%) | 0.32^3^ |
| eGFR, median (IQR) | 93 (76 -109) | 94 (77-111) | 93 (76-106) | 0.51^1^ |
| Frailty | 5 (2.1%) | 1 (0.8%) | 4 (3.3%) | 0.37^2^ |
| LMS | 2 (0.8%) | 0 (0%) | 2 (1.7%) | 0.50^2^ |
| LMS/LAD | 114 (48%) | 51 (43%) | 63 (53%) | 0.12^3^ |
| Multivessel PCI | 10 (4.2%) | 5 (4.2%) | 5 (4.2%) | >0.99^2^ |
| Vessel diameter, mean ± SD mm | 3.32 ± 0.55 | 3.20 ± 0.60 | 3.45 ± 0.46 | **<0.001^1^** |
| Lesion length, median (IQR) mm | 25 (20-30) | 25 (20-30) | 25 (20-30) | 0.70^1^ |
| Vessel diameter >3mm | 196 (82%) | 87 (73%) | 109 (92%) | **<0.001^3^** |
| Bifurcation disease | 100 (42%) | 50 (42%) | 50 (42%) | >0.99^3^ |
| True bifurcation | 23 (9.6%) | 12 (10%) | 11 (9.2%) | 0.83^3^ |
| Heavy calcification | 51 (21%) | 24 (20%) | 27 (23%) | 0.64^3^ |
| Fluoroscopy time (min) | 9.1 (6.6-12.9) | 8.6 (6.3-11.1) | 10.4 (7.1-14.1) | **0.03**^1^ |
| Contrast volume (ml) | 120 (100-150) | 115 (100-150) | 120 (100-150) | 0.33^1^ |
| PRE-TIMI flow | | | | |
| TIMI 0-1 | 180 (75) | 89 (74) | 91 (76) | 0.68^3^ |
| TIMI 2-3 | 60 (25) | 31 (26) | 28 (24) |  |
| POST-PCI TIMI flow |  |  |  |  |
| TIMI 0-1 | 2 (0.8) | 1 (0.8) | 1 (0.8) | >0.99^2^ |
| TIMI 2-3 | 238 (99.2) | 119 (99.2) | 119 (99) |  |
| Coronary dissections |  | | | 0.35^2^ |
| No angiographic evidence | 166 (69) | 87 (73) | 79 (66) |  |
| Type A | 37 (15) | 19 (16) | 18 (15) |  |
| Type B | 36 (15) | 14 (12) | 22 (18) |  |
| Acuity score, median (IQR) | 17 (12-23) | 17 (12-22) | 16 (12-23) | 0.82^1^ |

Supplementary table 2 indicates the baseline patient and angiographic characteristics in the propensity score matched DCB cohort.

Abbreviations: PVD: peripheral vascular disease, PCI: percutaneous coronary intervention, CABG: coronary arterial bypass graft, IHD: ischaemic heart disease, COPD: chronic obstructive pulmonary disease, eGFR: estimated glomerular filtration rate, LMS: left main stem, LAD: left anterior descending

^1^Wilcoxon rank sum test, ^2^Fisher’s exact test, ^3^Pearson’s Chi-squared test, ^4^Wilcoxon rank sum exact test
